# Genome-Wide Identification and Hormone-Induced Expression Analysis of the Anthocyanidin Reductase Gene Family in Sainfoin (*Onobrychis viciifolia* Scop.)

**DOI:** 10.3390/ijms262311256

**Published:** 2025-11-21

**Authors:** Yuqing Hu, Guangzhi Jiang, Jiayin Wang, Huan He, Lele Liu, Pingping Du, Hongbin Li, Fei Wang, Quanliang Xie

**Affiliations:** Xinjiang Production and Construction Corps Key Laboratory of Oasis Town and Mountain-Basin System Ecology, Key Laboratory of Xinjiang Phytomedicine Resource Utilization, Ministry of Education, College of Life Sciences, Shihezi University, Shihezi 832003, China; hyq979823109@163.com (Y.H.); jiangguangzhi2024@163.com (G.J.); wjyinee@163.com (J.W.); he_huan026@163.com (H.H.); lzjl6774@163.com (L.L.); dopingping@126.com (P.D.); lihb@shzu.edu.cn (H.L.)

**Keywords:** sainfoin, anthocyanidin reductase, ABA, MeJA, cytoplasm

## Abstract

Sainfoin (*Onobrychis viciifolia* Scop.) is an important legume forage. Its anthocyanidin reductase (ANR) catalyzes the conversion of anthocyanins to epicatechins. This conversion reaction is not only a key step in the biosynthesis of proanthocyanidins (PAs) but also directly influences both forage quality and stress resistance. Here, we systematically identified 67 *ANR* gene family members in autotetraploid sainfoin for the first time. Using bioinformatics approaches, we analyzed gene structure, conserved domains, motifs, and cis-regulatory elements of the identified *ANR* genes. In this study, phylogenetic analysis revealed that the *ANRs* clustered into 11 distinct clades, with genes within the same clade predominantly originating from closely related species within the same family. Significant collinearity with *Arabidopsis thaliana*, *Glycine max*, *Cicer arietinum*, and *Medicago truncatula* further revealed the conserved evolutionary path of this gene family. RT-qPCR analysis showed differential expression patterns of *OvANRs* in root, stem, and leaf tissues. For instance, *OvANR19* was significantly induced by abscisic acid (ABA) and methyl jasmonate (MeJA), with its expression upregulated by 79.7-fold and 3.8-fold in roots and by 16.2-fold and 31.3-fold in leaves. Furthermore, subcellular localization analysis confirmed that representative ANR proteins were localized in the cytoplasm. This study lays a foundation for molecular breeding aimed at enhancing stress resistance and forage quality in sainfoin.

## 1. Introduction

Sainfoin (*Onobrychis viciifolia* Scop.) is a perennial leguminous forage that is valued for its prominent drought tolerance, nitrogen-fixing capacity, and nutritional value [1,2,3]. It is thus considered to play a strategically significant role in ecological restoration and animal husbandry development in the arid regions of northwest China [4,5]. Furthermore, beyond being a high-quality forage resource, it stabilizes soil through its extensive root system and substantially improves soil fertility via symbiotic nitrogen fixation with rhizobia [6,7]. More importantly, the proanthocyanidins (PAs) enriched in sainfoin leaves effectively inhibit excessive degradation of ruminal proteins in ruminants, significantly improve nitrogen utilization efficiency, and prevent bloat occurrence. This characteristic makes sainfoin an ideal model for investigating tannin metabolism mechanisms in legumes [8,9,10]. Sainfoin is an autotetraploid species, whose genome doubling not only increases the complexity of systematic gene identification and functional characterization [11], but has also been associated with significant expansion of gene families in the PA biosynthetic pathway. This expansion has been confirmed to be directly correlated with increased gene expression levels and elevated PA content [12]. Particularly, the molecular mechanisms regulating PA synthesis, particularly the structural characteristics and functional networks of anthocyanidin reductase (ANR) at the genomic level, have not yet been systematically elucidated. Furthermore, the gene copy number variation, expression divergence, and functional redundancy under its tetraploid nature remain uncharacterized. It is noteworthy that abiotic stresses driven by climate change are severely constraining sainfoin productivity and persistence [13,14]. As plants typically rely on hormone signaling pathways to activate stress-responsive gene expression for survival [15]. Therefore, elucidating the molecular basis of its stress adaptation is crucial for the genetic improvement of sainfoin.

PAs synthesized through the flavonoid pathway serve as efficient antioxidants that scavenge reactive oxygen species (ROS), protect cellular membrane integrity, and alleviate oxidative damage [16,17,18,19], thereby forming a crucial physiological foundation for sainfoin’s resistance to abiotic stresses such as drought. Specifically, ANR, encoded by the *ANR* gene family, catalyzes the conversion of anthocyanidins to epicatechins, which constitutes a pivotal step in PA biosynthesis [20]. Studies have shown that in leguminous plants, *ANR* genes directly regulate PAs accumulation and stress resistance [21,22]. Currently, *ANR* genes have been extensively studied in diverse plant species, including *Camellia sinensis*, *Litchi chinensis*, *Malus pumila*, *Carthamus tinctorius*, and *Punica granatum* [23,24,25,26,27]. For example, 51 *ANR* members were identified in the litchi genome, among which the expression of *LITCHI029356.m1* showed a significant negative correlation with anthocyanin accumulation in the pericarp, and its function in inhibiting coloration was confirmed through heterologous expression in tobacco [24]. In the tea plant, *CsANR* catalyzes the formation of an unstable catechin carbocation intermediate, which serves as a crucial substrate for PAs polymerization; transgenic tobacco experiments confirmed that *CsANR* overexpression significantly suppressed anthocyanin biosynthesis genes while enhancing PAs accumulation [23]. Similarly, overexpression of apple *MdANR* was shown to significantly suppress the expression of chalcone isomerase (*CHI*) and dihydroflavonol 4-reductase (*DFR*) genes, thereby reducing the diversion of anthocyanin precursors and ensuring efficient PAs synthesis [25]. Another study successfully generated novel rice germplasm enriched with PAs and exhibiting high antioxidant capacity by expressing a grapevine *ANR* gene in rice under the control of an endosperm-specific promoter [28]. Furthermore, heterologous expression of mulberry *MnANR* and *MnLAR* genes in tobacco enhanced resistance to *Botrytis cinerea* compared to wild-type plants, with transgenic lines exhibiting alleviated disease symptoms [29]. However, systematic characterization of the *ANR* gene family remains lacking in leguminous forages, particularly in the polyploid species sainfoin. While the functions of *ANR* have been progressively elucidated in various plants, its mechanism of action in sainfoin has yet to be explored.

Plant hormones coordinately regulate stress responses and flavonoid biosynthesis [30,31,32]. During sainfoin’s adaptation to abiotic stresses such as drought, the abscisic acid (ABA) and methyl jasmonate (MeJA) signaling pathways play pivotal roles and are closely associated with the development of its stress resistance. Under stress conditions, the accumulation of ABA inhibits PP2C, thereby simultaneously relieving the suppression of both the ABA signaling pathway and the SnRK1 energy signaling pathway, which leads to their coordinated activation and collectively enhances the plant’s stress response [33]. MeJA, on the other hand, substantially boosts the synthesis and accumulation of flavonoids in *Dendrobium* pseudobulbs by regulating the expression of specific *DoMYCs*, revealing a ‘MeJA-MYC-flavonoid’ regulatory circuit [34]. It is noteworthy that complex cross-talk exists between the ABA and JA signaling pathways, which may precisely regulate metabolic flux [35,36]. In eggplant, *SmMYB5* acts as a key integrator that links jasmonate and light signaling pathways to regulate anthocyanin biosynthesis under low light stress [37]. However, functional differentiation exists between the two hormones: ABA primarily triggers osmoregulatory responses, while MeJA preferentially mediates the synthesis of defensive compounds [38,39]. Nevertheless, as an important forage, the specific response mechanisms of sainfoin *ANR* gene family members to ABA/MeJA signaling remain unclear. Whether expression patterns and functional differentiation exist among different members, and how they synergistically regulate PAs biosynthesis to enhance plant stress resistance under the autotetraploid background, constitute pressing scientific questions that require urgent investigation.

Furthermore, by utilizing published transcriptome data, we investigated the differential expression patterns of key members across different tissues under ABA and MeJA treatments to elucidate their functional specialization. The subcellular localization of representative members was also determined. The findings of this study provide crucial genetic resources and a theoretical foundation for deciphering the *OvANR*-mediated stress tolerance mechanisms in sainfoin and advancing molecular breeding for enhanced stress resistance.

## 2. Results

### 2.1. Identification of the OvANR Gene Family in Sainfoin

A total of 67 sainfoin genes encoding proteins with complete ANR domains were identified through a combination of homology-based searches, Hidden Markov Model (HMM) analysis, and conserved and domain validation techniques. These genes were systematically designated *OvANR1* to *OvANR67* according to their relative positions on the chromosomes (Supplementary Table S1). Bioinformatic analysis unveiled significant polymorphism among the *OvANR* family members. The encoded proteins exhibited a length range of 99 to 504 amino acids, with *OvANR30* being the longest (504 aa) and *OvANR48* the shortest (99 aa). The predicted molecular weights ranged from 10.51 to 56.83 kDa, and isoelectric points (pI) ranged from 4.84 to 9.00, indicating a range from acidic to basic proteins. Concurrently, Hydropathy analysis showed that 58 members (with an average GRAVY of −0.383) exhibited hydrophobic characteristics, while the remaining 9 members (with an average GRAVY of 0.227) were hydrophilic. Subcellular localization prediction revealed a distinct compartmentalization pattern for this family. The distribution analysis showed that 56 members (83.58%) were localized to the cytoplasm, 6 (8.96%) to the chloroplasts, 3 (4.48%) to the plasma membrane, and 2 (2.99%) to the mitochondria (Supplementary Table S1). This cytoplasm-predominant distribution pattern suggests that the *OvANR* family may primarily be involved in fundamental cellular processes within the cytoplasm, such as metabolic regulation, biosynthesis, or signal transduction.

### 2.2. Evolutionary Relationship, Conserved Domains, Motif Composition, and Gene Structure of OvANRs in Sainfoin

**Figure 1:**
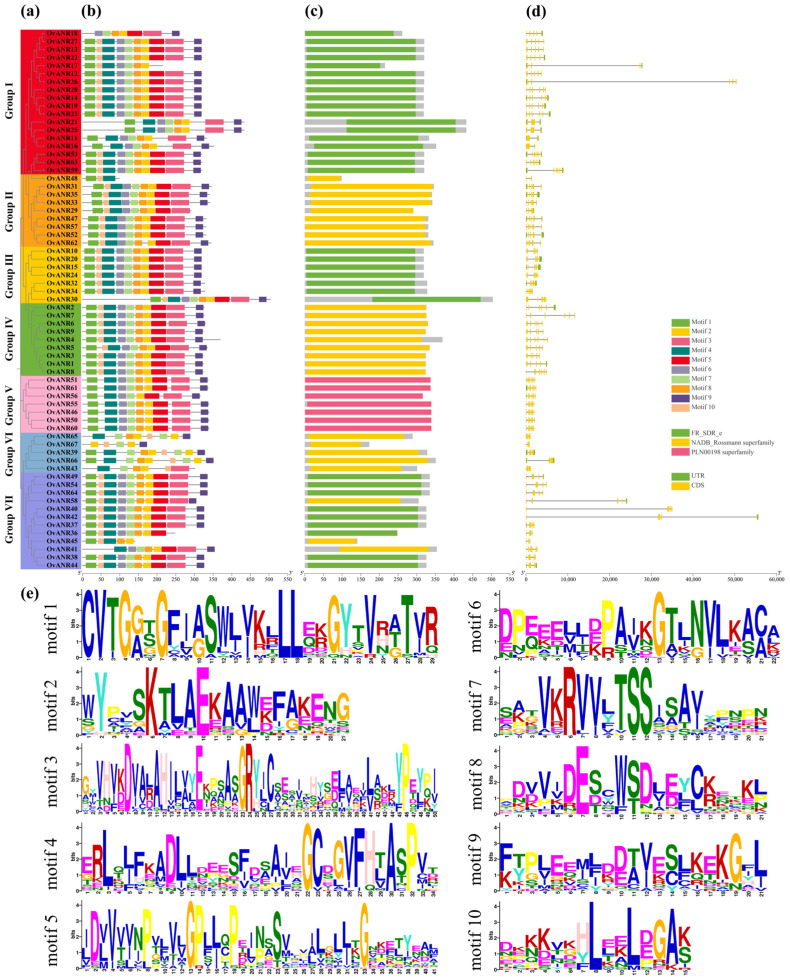
Evolutionary Relationship, Conserved Domains, Motif Composition, and Gene Structure of the OvANR family proteins in sainfoin. (**a**) Evolutionary tree of the *OvANR* gene family constructed using the neighbor-joining (NJ) method, with different colors representing distinct subfamilies. (**b**) Conserved motifs visualized by TBtools software v2.326, where boxes of different colors represent distinct motifs. A maximum of ten motifs is displayed. (**c**) Conserved domains in *OvANR* genes, including FR_SDR_e, NADB_Rossmann superfamily, and PLN01988 superfamily. (**d**) where green rectangles represent 5′-UTR and 3′-UTR, yellow rectangles represent CDS, and black lines represent introns. (**e**) Top ten conserved motifs identified in OvANR peptides.

To illustrate the structural composition of the *OvANR* genes, a gene structure map was constructed utilizing the genome and annotation files. This map visually represents the distribution of untranslated regions (UTRs), coding sequences (CDSs), and introns (Figure 1). Comparative analysis of the number and position of exons and introns across the 67 *OvANR* genes revealed considerable variation in their gene structures. All genes contained varying numbers of exons. Among these, 33 genes contained UTRs, while the remaining 34 lacked them. All 67 genes contained introns, with their numbers distributed as follows: 3 genes (4.48%) contained 1 intron, 8 (11.94%) contained 2, 10 (14.93%) contained 3, 13 (19.40%) contained 4, 31 (46.27%) contained 5, and 2 genes (2.98%) contained 6 introns. To further substantiate the structural characteristics of the OvANR proteins, analysis using the MEME tool indicated that *OvANRs* retained numerous conserved motifs during evolution. The results display the distribution of 10 highly conserved motifs identified across the 67 ANR protein sequences (Figure 1). Each family member contained between 5 and 10 conserved motifs. Specifically, motif 1 was predominantly located at the N-terminal region, while motif 9 was primarily concentrated at the C-terminal region.

### 2.3. Multiple Sequence Alignments, Secondary and Tertiary Structures of the OvANR Proteins in Sainfoin

Specifically, multiple sequence alignment (Supplementary Figure S5) revealed that among the 67 *OVANR* members, all except five—*OvANR18*, *OvANR41*, *OvANR43*, *OvANR65*, and *OvANR67*—contain the conserved NADP-binding site, while all but one, *OvANR48*, possess the conserved active site. In addition, several regions with sequence conservation above 70% and 50% have been observed. Collectively, these results indicate that although not completely conserved, these key motifs remain highly conserved throughout the evolution of OvANR proteins. Secondary structure analysis of OvANR proteins (Supplementary Table S2) revealed that they primarily consist of α-helix (27.57–52.98%), β-turn (6.53–27.02%), extended strand (11.29–26.25%), and random coil (16.06–37.76%). Notably, the proportion of random coils exhibited the most significant variation among different proteins, suggesting potential diversity in structural flexibility among family members. Further prediction of the tertiary structures (Supplementary Figure S1) indicated that these proteins contain typical structural elements, including α-helices, β-sheets, and random coils. The predictive models also revealed that the core scaffold of OvANR proteins is mainly formed by α-helix and β-turn, with these regular structural domains interconnected by flexible random coil regions, collectively assembling into their specific functional domains. This structural organization pattern aligns with the typical topological features of many functional proteins, implying a close relationship between domain formation and functional execution.

### 2.4. Cis-Acting Element Analysis of OvANR Gene Family in Sainfoin

**Figure 2:**
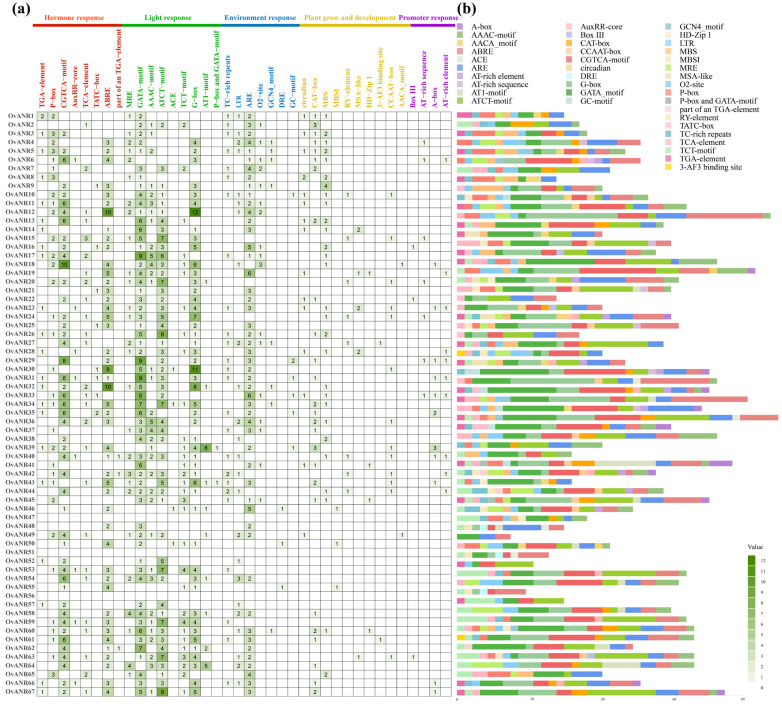
Cis-acting elements in the promoters of the *ANR* gene family in Sainfoin. (**a**) Classification and heatmap analysis of all cis-acting elements in the promoters of *OvANRs* based on the PlantCARE database, where the color intensity in the heatmap represents the quantity of each type of element. (**b**) In the stacked chart, different colors indicate the number of each cis-acting element type in individual *OvANR* genes.

Cis-acting elements are binding sites for transcription factors and other functional motifs that play crucial roles in regulating transcriptional initiation [40]. In this study, we performed a systematic analysis of Cis-acting elements within the 2000 bp promoter regions upstream of the *OvANR* genes using the PlantCARE database, with results visualized in Figure 2. The analysis revealed that this region contains a substantial number of regulatory elements closely associated with plant hormone responses and environmental stress responses, including jasmonic acid-responsive elements (CGTCA-motif and G-box), abscisic acid-responsive elements (ABRE), gibberellin-responsive elements (P-box and TATC-box), salicylic acid-responsive elements (TCA-element and TGA-element), auxin-responsive elements (AuxRR-core), anaerobic-responsive elements (ARE and GC-motif), low-temperature-responsive elements (LTR), drought- and high-salinity-responsive elements (MBS), and light-responsive elements (ACE and BOX-III). These observations suggest that the expression of *OvANR* genes is likely co-regulated by multiple endogenous hormonal signals and external environmental stimuli. Furthermore, the distinct cis-acting elements compositions among different *OvANR* members suggest potential functional specificity, underscoring the importance of this gene family in normal plant development. Notably, the promoter of *OvANR19* contains five ABREs and three G-box elements, suggesting its expression might be induced by ABA and MeJA treatments, and implying potential regulation by ABRE-binding proteins and MYC2 transcription factors. Collectively, these results highlight the significant role of *OvANR* genes in plant growth, development, and adaptation to complex environmental changes.

### 2.5. Evolutionary Analysis of OvANR Proteins

**Figure 3:**
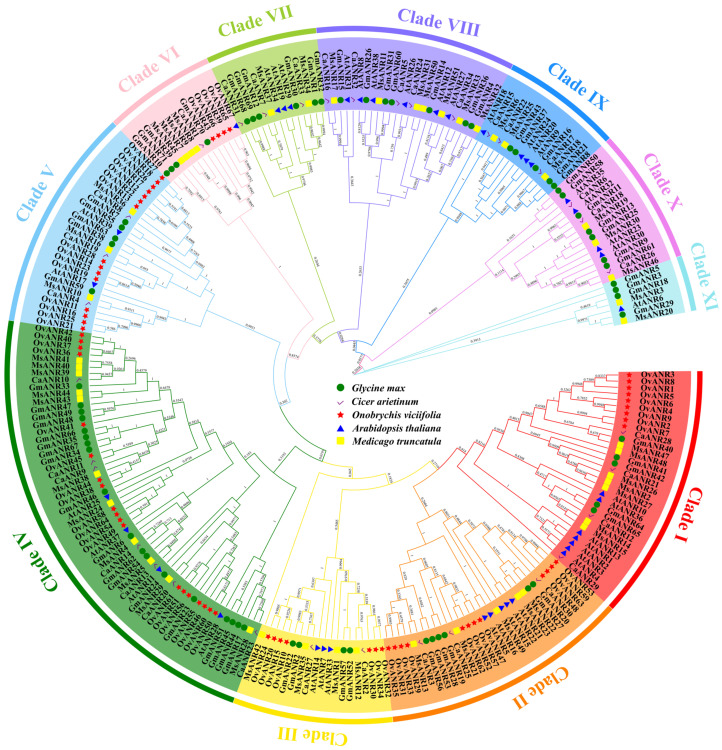
Phylogenetic tree of ANR proteins from *Onobrychis viciifolia*, *Arabidopsis thaliana*, *Glycine max*, *Cicer arietinum*, and *Medicago truncatula*. The tree was constructed using the neighbor-joining method with 1000 bootstrap replicates. Species are marked with distinct symbols: green circles for *Glycine max*, purple checkmarks for *Cicer arietinum*, red stars for *Onobrychis viciifolia*, blue triangles for *Arabidopsis thaliana*, and yellow squares for *Medicago truncatula*.

To elucidate the evolutionary relationships within the *ANR* gene family in sainfoin, this study integrated *ANR* genes from the model plant *Arabidopsis thaliana* (39 genes) and leguminous species, including *Glycine max* (71 genes), *Cicer arietinum* (34 genes), and *Medicago truncatula* (51 genes), along with the 67 identified *OvANR* genes from sainfoin, to construct a phylogenetic tree using the neighbor-joining method in MEGA-X v10.2.6 (Figure 3). The results revealed a marked uneven distribution of *OvANR* genes across subfamilies, with subfamily IV containing 19 *OvANR* genes (accounting for 32.2% of the total members in this subfamily) and forming the largest evolutionary clade. However, subfamilies VII–XI were entirely devoid of *OvANR* genes. These findings suggest that the *ANR* gene family in *O. viciifolia* may have undergone functional simplification-type evolution.

### 2.6. Chromosomal Localization and Collinearity Analysis of OvANR Genes

**Figure 4:**
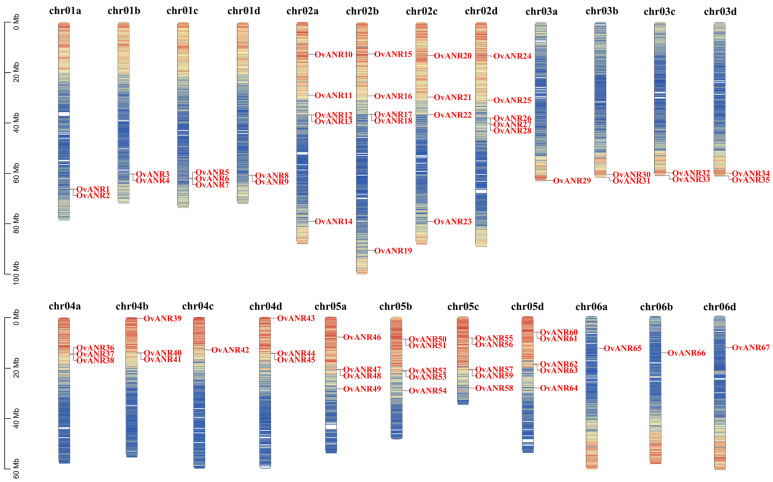
Chromosomal localization of *OvANR* genes. The 23 chromosomes of sainfoin are labeled Chr01a to Chr06d. The scale on the left indicates chromosomal length (Mb). Chromosome numbers are shown in black, while *OvANR* genes are marked in red. The color gradient from red to blue represents the gene density from high to low.

The 67 genes of the sainfoin *ANR* gene family were unevenly distributed across 23 chromosomes (Figure 4). The analysis revealed a distinct non-random genomic distribution pattern for this gene family. Specifically, chromosomes Chr03a, Chr04c, Chr06a, Chr06b, and Chr06d each carried only a single *OvANR* member. In comparison, chromosomes Chr01a, Chr01b, Chr01d, Chr03b, Chr03c, and Chr03d harbored two *OvANR* genes each. A higher density was observed on chromosomes Chr01c, Chr04a, Chr04b, and Chr04d, each of which contains three members. Chromosomes Chr02c and Chr05a each carried four *OvANR* genes. Notably, the greatest concentration of *OvANR* family members was found on chromosomes Chr02a, Chr02b, Chr02d, Chr05b, Chr05c, and Chr05d, each accommodating five genes, highlighting the significance of these chromosomal regions in the organization and potential evolutionary history of the *OvANR* gene family.

**Figure 5:**
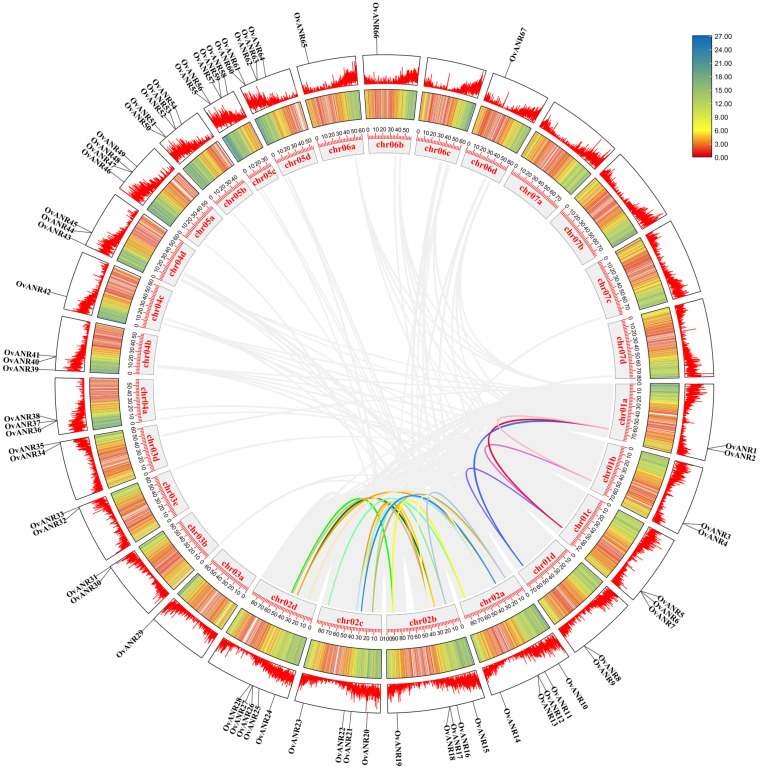
Collinearity analysis of *ANR* genes in sainfoin. The inner circle’s light gray lines represent all collinear gene pairs within the species, while colored lines highlight tandem duplication relationships among *OvANR* genes. Chromosome names and lengths are labeled in the inner circle. The middle and outer circles display gene density distributions, with blue indicating high-density regions and red indicating low-density regions.

Intraspecific collinearity analysis of the *ANR* gene family in sainfoin revealed that among the 67 family members, 24 genes exhibit collinear relationships (Figure 5). These results suggest that these *OvANR* genes may possess similar functional properties. Additionally, the gray lines in the figure further illustrate collinearity among other genes across the genome, reflecting their evolutionary conservation. In this study, we identified 21 collinear gene pairs involving 24 *OvANR* genes on chromosomes Chr01a, Chr01b, Chr01c, Chr01d, Chr02a, Chr02b, Chr02c, and Chr02d (Figure 4). No tandem duplication events observed. Notably, all genes undergoing segmental duplication belong to the same subfamily. For instance, *OvANR1* and *OvANR3* from subfamily IV are a pair of homologous genes generated by segmental duplication. Similarly, *OvANR12* and *OvANR17* from subfamily I are also homologous genes. Furthermore, we analyzed segmental duplication events among the 67 *OvANR* genes, identifying 24 homologous loci and 21 segmental duplication gene pairs. These pairs include: *OvANR1/OvANR3*, *OvANR2/OvANR7*, *OvANR1/OvANR8*, *OvANR3/OvANR5*, *OvANR4/OvANR6*, *OvANR5/OvANR8*, *OvANR12/OvANR17*, *OvANR10/OvANR15*, *OvANR14/OvANR19*, *OvANR11/OvANR21*, *OvANR12/OvANR22*, *OvANR14/OvANR23*, *OvANR10/OvANR20*, *OvANR10/OvANR24*, *OvANR11/OvANR25*, *OvANR16/OvANR21*, *OvANR17/OvANR22*, *OvANR15/OvANR20*, *OvANR17/OvANR26*, *OvANR19/OvANR28*, and *OvANR16/OvANR25*. Based on their subfamily affiliations, 11 of these 21 segmental duplication pairs belong to subfamily I, 6 to subfamily IV, and 1 to subfamily III. The high concentration of segmental duplication genes in subfamily I suggests that members of this subfamily may have special functional importance or have undergone functional specialization related to environmental adaptation.

The Ka/Ks ratio was used to elucidate the evolutionary processes and selective pressures acting on *OvANRs*. A Ka/Ks value of 1 indicates neutral selection, <1 indicates purifying selection, and >1 indicates positive selection [41]. The results showed that the Ka/Ks values for the segmental duplications ranged from 0.0203 to 0.6616, with an average value of 0.3375 (Supplementary Table S3). All Ka/Ks values for the duplicated pairs were less than 1, indicating that the *OvANR* genes have predominantly undergone purifying selection during evolution.

**Figure 6:**
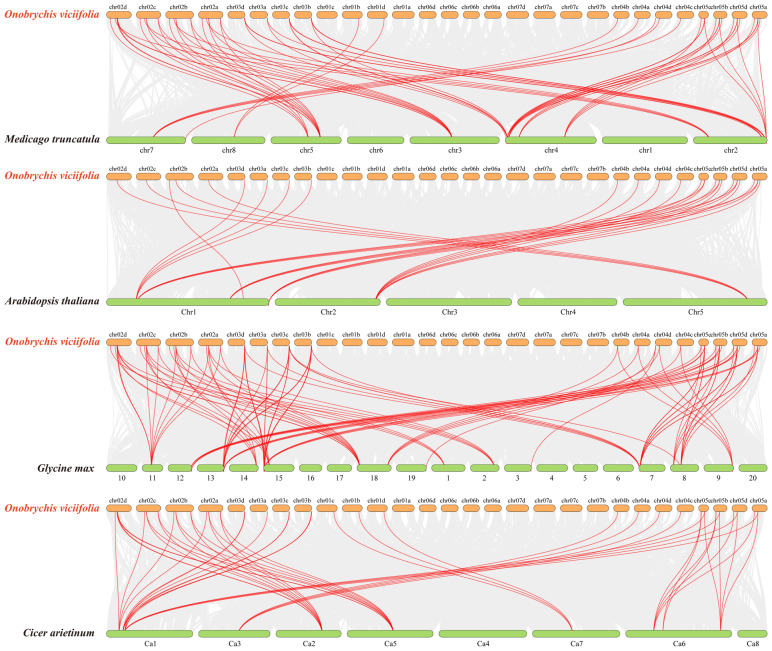
Collinearity analysis of *ANR* genes between *Onobrychis viciifolia* and *Medicago truncatula*, *Arabidopsis thaliana*, *Glycine max*, and *Cicer arietinum*. Light gray lines represent all collinear gene pairs across species, while red lines specifically highlight the collinear relationships of *ANR* gene pairs. Species names are labeled on the left side of the figure. Orange bars denote the chromosomes of *O. viciifolia*, and green bars represent the chromosomes of *M. truncatula*, *A. thaliana*, *G. max*, and *C. arietinum*.

To gain deeper insights into the potential evolutionary relationships of the *OvANR* gene family, we systematically analyzed genomic collinearity between sainfoin and the model plant *Arabidopsis thaliana*, as well as three closely related legume species: *Glycine max*, *Cicer arietinum*, and *Medicago truncatula* (Figure 6). This analysis identified multiple genes exhibiting collinearity with the sainfoin *ANR* genes. Notably, the degree of collinearity was substantially stronger among legume species compared to the cross-family comparison. Specifically, *Glycine max*, also a legume, displayed the highest number of collinear gene pairs with sainfoin. In contrast, *Arabidopsis thaliana*, a member of the Brassicaceae family, showed the fewest collinear genes. Specifically, the number of collinear gene pairs between sainfoin and the other species varied: 28 pairs with *Arabidopsis thaliana*, 50 pairs with *Cicer arietinum*, 57 pairs with *Medicago truncatula*, and the highest number, 97 pairs, with *Glycine max* (Supplementary Table S4). This indicates a higher degree of evolutionary conservation between *Onobrychis viciifolia* and *Glycine max*. Furthermore, no collinear gene pairs were found between sainfoin chromosomes Chr01a, Chr06a, Chr06b, Chr06c, Chr06d, Chr07a, Chr07b, Chr07c, and Chr07d and any chromosomes of the other four plant species. This may be related to the functional specificity of *ANR* genes in these regions. These results provide valuable references for analyzing the genetic relationships and gene functions among these species.

### 2.7. The Expression Pattern Analysis of OvANR Genes in Sainfoin

**Figure 7:**
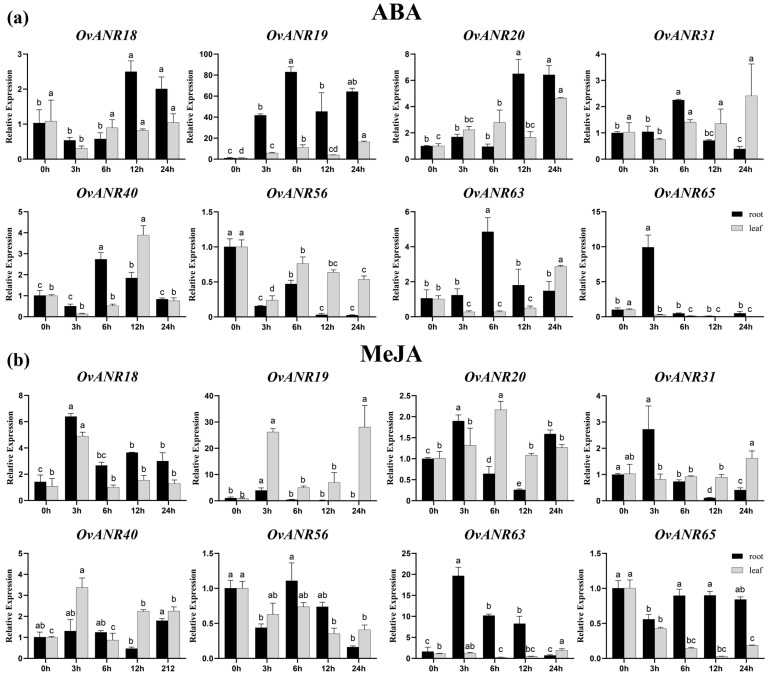
Expression analysis of eight selected *OvANR* genes in roots and leaves under ABA and MeJA treatments. (**a**) Relative expression levels of *OvANR* at 0 h, 3 h, 6 h, 12 h, and 24 h post-ABA treatment. (**b**) Relative expression levels of *OvANR* at 0 h, 3 h, 6 h, 12 h, and 24 h post-MeJA treatment. Data were normalized using the Actin gene as an internal reference. Error bars represent standard deviations. Different letters indicate significant differences determined by Duncan’s multiple range test (*p* < 0.05).

Based on published transcriptome data under cold and drought stress, we compiled and analyzed the expression profiles of all 67 *OvANR* family members (Supplementary Figure S3). To further investigate the regulatory roles of abscisic acid (ABA) and methyl jasmonate (MeJA) on key stress-responsive genes, we selected eight representative genes for in-depth study based on their significantly up-regulated expression patterns under cold and drought stress. Subsequently, one-month-old sainfoin plants from different tissues (roots, stems (Supplementary Figure S4), and leaves) were treated with exogenous ABA and MeJA. The relative expression levels of these eight *OvANR* members in different tissues and under different hormone treatments were systematically analyzed at 0, 3, 6, 12, and 24 h time points using RT-qPCR (Figure 7).

Under ABA treatment, the expression timing of different genes varied across tissues. In the roots, *OvANR65* responded most rapidly to ABA, reaching its peak expression level at 3 h, after which the expression gradually decreased. In contrast, the expression of *OvANR56* dropped to its lowest level at 3 h, showed a brief increase at 6 h, and then decreased again, remaining below the control level. Apart from the aforementioned genes, the peak expression of the remaining genes in the roots mostly occurred within 6–12 h. In the leaves, apart from the consistently down-regulated *OvANR56* and *OvANR65*, *OvANR40* reached its peak expression at 12 h, while the remaining genes all reached their highest expression levels at 24 h. Overall, the expression peaks in the roots generally occurred earlier than those in the leaves.

Under MeJA treatment, the response pattern of genes in the roots was similar to that under ABA treatment. Except for *OvANR40* (peaking at 24 h) and *OvANR56* (peaking at 6 h), the other genes all reached their peak expression at 3 h. Meanwhile, *OvANR65* was consistently down-regulated in both roots and leaves. In the leaves, apart from the persistently suppressed expression of *OvANR56* and *OvANR65*, *OvANR18* and *OvANR40* reached their peaks at 3 h, indicating the most rapid response. The expression levels of the remaining genes were significantly higher than the control within 6–24 h. These results further indicate that root tissues respond more rapidly to exogenous hormone application compared to leaves.

Expression analysis revealed that, compared to the corresponding control groups, most of the selected *OvANR* genes showed up-regulated expression trends in response to both ABA and MeJA treatments. Notably, *OvANR19* was significantly induced by both ABA and MeJA. In roots, its expression was up-regulated by 79.7-fold and 3.8-fold. Respectively, while in leaves, it was up-regulated by 16.2-fold and 31.3-fold. In contrast, the expression of *OvANR56* and *OvANR65* was primarily exhibited in down-regulation trends, with distinct tissue-specific patterns. In leaves, the expression of both *OvANR56* and *OvANR65* was down-regulated by both hormones. In roots, however, the expression of *OvANR56* was down-regulated only by ABA, and the expression of *OvANR65* was down-regulated only by MeJA, indicating a tissue-preferential response to hormone signals. Furthermore, these results suggest that the *OvANR* gene family is likely involved in the ABA and MeJA metabolic pathways.

### 2.8. Subcellular Localization of OvANRs

**Figure 8:**
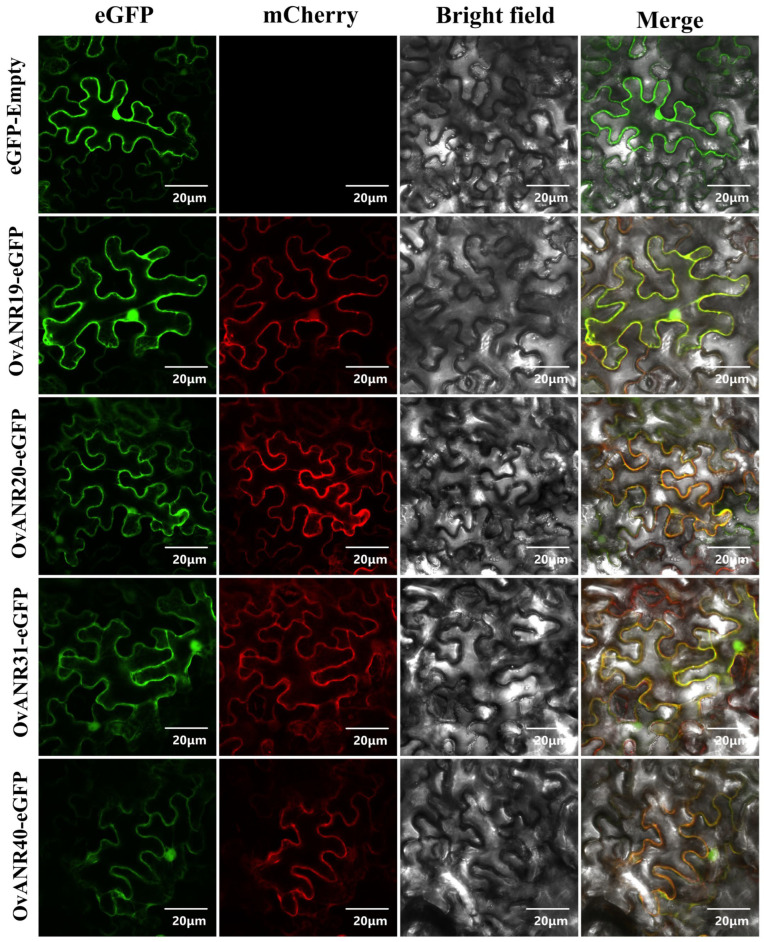
Subcellular localization of OvANR proteins in *Nicotiana benthamiana* leaves. The pCAMBIA1300-35S-eGFP (empty vector control) and pCAMBIA1300-35S-OvANR-eGFP (OvANR-eGFP fusion proteins) constructs were transiently expressed in epidermal cells of *N. benthamiana* leaves via *Agrobacterium*-mediated transformation. The cytoplasmic marker (pCAMBIA1300-35S-mCherry-NOS) was labeled with the mCherry fluorescent protein. The images show the green fluorescent channel (eGFP), red fluorescent channel (mCherry), bright field, and merged eGFP/mCherry channels. In the merged images, yellow colour indicates co-localization of the green and red signals.

To determine the precise subcellular localization where *OvANRs* exert their function, we selected four key genes (*OvANR19*, *OvANR20*, *OvANR31*, and *OvANR40*) that were significantly upregulated in the cold-drought stress transcriptome data (Supplementary Figure S3) as well as upon ABA and MeJA treatments (Figure 7) for subcellular localization analysis. We employed a transient transformation technique in *Nicotiana benthamiana*. Recombinant vectors (35S-eGFP) fused with *OvANR19*, *OvANR20*, *OvANR31*, by constructing recombinant vectors (35S::OvANR-eGFP) fusing these genes with eGFP, using the empty eGFP vector (35S::eGFP) as a control (Figure 8). Observation under a confocal laser scanning microscope revealed that the green fluorescent signals (eGFP) of all four fusion proteins co-localized with the red fluorescent signal of the cytoplasmic marker mCherry, indicating their primary localization in the cytoplasm. This observation aligns with the predictions from the CELLO online subcellular localization tool (Supplementary Table S1).

## 3. Discussion

Anthocyanin reductase (ANR), a key enzyme in the flavonoid metabolic pathway, catalyzes the reduction of anthocyanidins to 2,3-cis-flavan-3-ols, primarily epicatechin, which subsequently forms proanthocyanidins (PAs) [42]. PAs not only reduce rumen bloat in ruminants but also serve as effective antioxidant barriers, acting as important defensive mechanisms against oxidative stress and maintaining cellular membrane integrity [16,43,44]. Furthermore, ANR serves not only as a key enzyme for PAs biosynthesis but also exerts feedback inhibition on the upstream anthocyanin synthesis pathway [22]. Although the importance of *ANR* genes in plant PAs synthesis has been widely recognized, research on ANR in sainfoin remains relatively limited. Therefore, this study performed a genome-wide identification of the *ANR* gene family in sainfoin based on publicly available genomic data [10]. A total of 67 *OvANR* genes were identified, and together with *ANR* genes from four representative species (*Arabidopsis thaliana*, *Glycine max*, *Cicer arietinum*, and *Medicago truncatula*), a phylogenetic tree comprising 262 *ANR* members was constructed, clustering into 11 distinct clades (Clade I–XI). Notably, *OvANR* genes are predominantly distributed in Clades I–VI (Figure 3), suggesting that the *ANR* gene family in sainfoin may have undergone specific evolutionary divergence during its adaptation.

Gene structural analysis demonstrated that the intron count in *OvANR* family members ranges from 1 to 5. Remarkably, *OvANR11*, *OvANR16*, and *OvANR48* each contain only a single intron and exhibit clustered genomic distribution, suggesting their origin from recent gene duplication events (Figure 1). Gene duplication serves as a crucial mechanism for gene family expansion and functional divergence, being especially prevalent in plant secondary metabolic gene families [45]. Conserved motif analysis identified 10 motifs, with most clades (I–VII) displaying highly conserved motif compositions, indicating close evolutionary relationships within the family. However, *OvANR45*, *OvANR48*, and *OvANR67* exhibited significant deviations from the core motif pattern, primarily lacking 5–7 motifs. Similar phenomena have been documented in plant secondary metabolism gene families, where motif loss correlates with altered substrate specificity [46]. Transmembrane helix prediction confirmed the absence of transmembrane domains in all *OvANR* family members (Supplementary Figure S2). Predicted protein structures further elucidate functional divergence mechanisms: secondary structures are dominated by α-helices forming a characteristic Rossmann fold core to ensure coenzyme binding (Supplementary Table S2). While tertiary structures remain highly conserved across most members, *OvANR45*, *OvANR48*, and *OvANR67* likely undergo significant structural variations due to motif loss (Supplementary Figure S1) [47]. The absence of the conserved NADP-binding site in several members, such as *OvANR18* and *OvANR41*, suggests their possible functional specialization, warranting further investigation into their biological roles (Supplementary Figure S5) [48].

This study identified 67 *ANR* genes in sainfoin, a number significantly higher than that commonly found in diploid legume plants. This large-scale expansion is likely closely related to the genomic characteristics of sainfoin as an autotetraploid, whose genomic complexity is substantially greater than that of its diploid relatives. Genome duplication resulting from polyploidization provides a fertile ground for gene family expansion. This tetraploid nature has led to gene number amplification, potentially providing genetic redundancy for metabolic pathways [49]. Meanwhile, gene amplification in sainfoin has been confirmed to be a key mechanism by which the expression of PAs biosynthetic pathway-related genes is enhanced, leading to increased PAs accumulation [12]. Notably, some *OvANR* genes (e.g., *OvANR29-35*) show positional conservation on homologous chromosomes and are concentrated in subtelomeric regions (Figure 4). The high sequence conservation of these genes indicates they have been under strong purifying selection during evolution, which may be crucial for maintaining the stability of the PAs biosynthesis pathway [50]. Intraspecific synteny analysis revealed significant conservation within its genome. The study found that 24 genes were involved in syntenic relationships, forming 21 syntenic pairs (Figure 5). This finding demonstrates that the genomic architecture has remained relatively stable throughout evolution, with the arrangement and linkage of some genes being conserved, suggesting these regions may be constrained by functional selective pressures. Similar genomic conservation has been observed in other legume species such as soybean indicating this phenomenon might be a common characteristic in legume evolution [51]. It is noteworthy that segmental duplication events were highly enriched in subfamily I (11 pairs), suggesting that members of this subfamily may have undergone expansion related to specific functional demands. Gene segmental duplication is a major driver of plant genome evolution and neofunctionalization [52]. The subset of paralogs created by WGD are homeologs. Homologous genes or genomic regions derived from their duplication within a lineage are paralogs. Homologous genes or genomic regions derived from the divergence of lineages are orthologs [53]. The large-scale expansion in sainfoin as an autotetraploid has generated a substantial number of homeologs. These homeologs may have undergone functional divergence during evolution. The 21 collinear gene pairs identified by intraspecific collinearity analysis are mainly distributed on corresponding chromosomes, such as Chr01a/01b/01c/01d and Chr02a/02b/02c/02d (Figure 5). This systematic collinearity pattern across homologous chromosome groups strongly suggests that the vast majority of these gene pairs are homeologs produced by the sainfoin autotetraploidization event [54]. Notably, these homeologs (e.g., *OvANR1/OvANR3*, *OvANR12/OvANR17*) have all been under strong purifying selection after duplication (all Ka/Ks values < 1) (Supplementary Table S3). This indicates they may maintain metabolic network stability through functional redundancy or have undergone subfunctionalization, collectively undertaking the complete function of the ancestral gene, thus being preserved during evolution. The most extensive collinear relationships were found between *Onobrychis viciifolia* and *Glycine max* (97 collinear pairs), far exceeding those with other species, confirming their relatively close phylogenetic relationship (Figure 6). These cross-species collinear gene pairs are orthologs, which likely inherited core biochemical functions from a common ancestor. Meanwhile, interspecific collinearity analysis revealed deep evolutionary homology between sainfoin and other legume species, indicating that *ANR* genes already existed in the legume ancestor and have evolved conservatively [55]. Despite long independent evolution, extensive genomic blocks between the legume species *Onobrychis viciifolia*, *Glycine max*, *Cicer arietinum*, *Medicago truncatula*, and the dicot model plant *Arabidopsis thaliana* still exhibit high conservation in gene content and order. These regions often contain crucial, functionally essential genes, such as those involved in basic metabolism, cell cycle regulation, and stress response [56].

ABA and MeJA, as pivotal phytohormones, extensively regulate plant growth, stress responses, and secondary metabolism [57,58,59]. Studies have shown that overexpression of *RrANR* enhances plant tolerance to oxidative stress by increasing ROS scavenging and modulating the ABA signaling pathway [60]. Exogenous MeJA treatment activates *ANR* expression via de-repression of the MdbHLH3-MdMYB complex by MdJAZ proteins, subsequently promoting PAs biosynthesis through direct binding and upregulation of *ANR* genes by MdMYB9/11 transcription factors [61]. Prior research indicates that ANR participates in hormone and stress responses. Thus, we treated different tissues of sainfoin with ABA and MeJA (Figure 7). In leaf tissues, most *OvANR* members exhibited upregulated expression except *OvANR56* and *OvANR65*, which were downregulated. In root tissues, MeJA specifically suppressed *OvANR65* expression, while ABA specifically suppressed *OvANR56* expression, suggesting that these two genes may be regulated by a common upstream inhibitor. Additionally, both hormones influenced *ANR* expression in stems, inducing upregulation of certain genes (Supplementary Figure S4). Cold-drought stress transcriptome data (Supplementary Figure S3) revealed significant divergence in the response patterns of *OvANR* family members. Genes upregulated under cold-drought stress (e.g., *OvANR19*, *OvANR31*) were also highly sensitive to ABA or MeJA treatments. Synergistic interactions between hormone signaling pathways in multi-stress responses have been documented in plants [62]. Such differential responses further support functional divergence rather than redundancy within the *ANR* gene family. This indicates that multiple genes can be significantly induced. Since proanthocyanidins are known to be effective antioxidants, the expansion and coordinated regulation of the *ANR* gene family are likely to enhance the oxidative stress tolerance of sainfoin. Cis-acting elements, as core regulatory sequences in gene promoters, play indispensable roles in coordinating these biological processes [63]. As expected, genes containing hormone-responsive elements such as ABRE and G-box (e.g., *OvANR19*, *OvANR18*, etc.) were generally significantly induced by ABA and MeJA treatments (Figure 2). Among them, *OvANR19* was strongly upregulated in both roots and leaves, suggesting it may be a direct downstream target of hormone signaling pathways. Notably, genes for which promoter analysis did not predict typical ABRE or G-box elements showed markedly different or weak hormone response patterns. For instance, expression profiling of *OvANR56* and *OvANR65* revealed that the former responded only weakly in MeJA-treated leaves, while the latter responded specifically to ABA treatment in roots. This finding is highly consistent with the bioinformatics predictions, indicating that the presence of core cis-regulatory elements in the promoter is likely a key molecular basis for driving hormone responses. Furthermore, the spatiotemporal dynamics of gene expression revealed additional complexity. The generally earlier response of genes in roots compared to leaves suggests that even when the same cis-elements are present, their regulatory effectiveness can be profoundly influenced by tissue-specific factors. Supporting this, studies in *Arabidopsis*, AREB/ABF transcription factors activate ABA-responsive genes via binding to ABREs [64]. ABA treatment significantly upregulates the expression of JA biosynthesis genes increasing JA levels, which leads to JAZ protein degradation and subsequent de-repression of MYC2 [65]. This suggests the potential existence of a synergistic regulatory module: under ABA signaling, AREB/ABF transcription factors can bind to ABREs, while under MeJA signaling, the MYC2 transcription factor can bind to G-box elements. These factors may form a complex transcriptional regulatory network that collaboratively and finely regulates the expression of the *ANR* gene. Such a synergistic regulatory module may play a critical role in sainfoin’s response to environmental stresses and hormonal signals. Finally, subcellular localization experiments confirmed that the selected ANR proteins are localized in the cytoplasm (Figure 8). This indicates that the proteins encoded by *ANR* genes likely perform their biological functions specifically within this subcellular compartment. It has been confirmed that *VaANR* is localized in the cytoplasm in grape. [66]. The four OvANR proteins localized in this study (OvANR19, OvANR20, OvANR31, and OvANR40) all exhibited significant upregulation under both cold-drought stress and phytohormone treatments, suggesting they may play a pivotal role in plant stress responses.

In summary, the large-scale expansion of the *ANR* gene family in sainfoin is likely a direct result of its genomic evolution as an autotetraploid. This expansion may not only provide the genetic basis for efficient proanthocyanidin biosynthesis but also enhance the metabolic plasticity and oxidative stress tolerance of sainfoin under complex environmental conditions through functional differentiation of some members. Based on these findings, this study provides a deeper and more systematic understanding of the *OvANR* gene family in sainfoin, laying an important foundation for future functional dissection.

## 4. Materials and Methods

### 4.1. Identification of the OvANR Gene Family and Analysis of Protein Physicochemical Properties in Sainfoin

The sainfoin genome sequence and annotation files were obtained from the National Genomics Data Center (NGDC, https://ngdc.cncb.ac.cn/, accessed on 3 October 2024) [12,55]. Initially, we retrieved the reported *ANR* genes from Arabidopsis thaliana [67,68] (AT1G61720, from The Arabidopsis Information Resource (TAIR), https://www.arabidopsis.org/, accessed on 3 October 2024) and confirmed the presence of the ANR conserved domain. Based on this domain, the complete sainfoin proteome was systematically screened using a Hidden Markov Model (HMM, PF01370) profile. Candidates identified preliminarily by HMMER v3.0 were subsequently validated for the integrity of their protein domains using the SMART (https://smart.embl.de/, accessed on 3 October 2024) and Pfam (http://pfam.xfam.org/, accessed on 3 October 2024) databases [69,70]. The NCBI CD-Search tool was also used to enhance the reliability of the validation [71] (https://www.ncbi.nlm.nih.gov/Structure/cdd/wrpsb.cgi, accessed on 3 October 2024). Ultimately, 67 sainfoin genes containing complete ANR domains were identified. The molecular weight, theoretical isoelectric point (pI), amino acid composition, and instability index for each protein were obtained using the ExPASy online server (http://www.expasy.org, accessed on 3 October 2024). Subcellular localization was predicted using CELLO [72] (https://cello.life.nctu.edu.tw/, accessed on 3 October 2024).

### 4.2. Evolutionary, Gene Structure, Conserved Domain, and Motif Analysis

Evolutionary, gene structure, and conserved domain analyses of the sainfoin *ANR* gene family were performed using the Gene Structure View (Advanced) module of TBtools software v2.326 [73]. Concurrently, conserved motifs within this family were analyzed using the online tool MEME [74] (https://meme-suite.org/meme/tools/meme, accessed on 3 October 2024).

### 4.3. Prediction of Secondary and Tertiary Structures

We compared the OvANR protein sequence using ClustalW online tool (https://www.genome.jp/tools-bin/clustalw, accessed on 3 October 2024) and visualized it with DNAMAN v6.0 [75]. The secondary structures of the OvANR proteins were analyzed using the SOPMA online tool [76] (https://npsa-pbil.ibcp.fr/cgi-bin/npsa_automat.pl?page=/NPSA/npsa_sopma.html, accessed on 3 October 2024). The tertiary structures were predicted using the SWISS-MODEL online platform [77] (https://swissmodel.expasy.org/interactive, accessed on 3 October 2024).

### 4.4. Cis-Acting Elements Analysis

The 2000 bp promoter sequences upstream of the start codons of sainfoin *OvANR* genes were extracted using the “Extract Promoter Sequences” module in TBtools software v2.326. Subsequently, the sequences were submitted to the PlantCARE database [78] (http://bioinformatics.psb.ugent.be/webtools/plantcare/html/, accessed on 3 October 2024). Following the initial identification of all potential elements, common basal eukaryotic elements (CAAT-BOX and TATA-BOX) as well as some unnamed or unannotated elements were excluded. Key cis-acting elements with potential regulatory functions were then filtered. The screening results were visualized using RStudio v4.5.0; a heatmap was generated to display the distribution density of each element, and a stacked bar plot was created to show the quantitative composition of different types of elements.

### 4.5. Phylogenetic Analysis

A phylogenetic tree was constructed using the neighbor-joining (NJ) method in MEGA-X software v10.2.6 [79]. The parameters were set as follows: the phylogenetic test was performed with 1000 bootstrap replicates, the substitution model was p-distance, and gaps/missing data were treated using the pairwise deletion method. Finally, the resulting phylogenetic tree was visualized and refined using the EvolView online tool (https://www.evolgenius.info/evolview/, accessed on 3 October 2024).

### 4.6. Chromosomal Location and Synteny Analysis

Chromosomal location analysis of the *OvANR* genes was performed using the “Gene Location Visualize” module in TBtools software v2.326. Furthermore, intraspecific synteny within this gene family and interspecific synteny between sainfoin and *Arabidopsis thaliana*, *Glycine max*, *Cicer arietinum*, and *Medicago truncatula* were analyzed using the “One Step MCScanX” module of the same software. Details of the relevant species are provided in Supplementary Table S6.

### 4.7. Cold and Drought Transcriptome Data

The raw transcriptome data used in this study were sourced from a previously published study under NCBI (https://www.ncbi.nlm.nih.gov, accessed on 3 October 2024) project number PRJNA553090. This data originated from a combined cold and drought stress experiment. The raw data were downloaded and converted to FASTQ format using SRA-Toolkit v2.9 (NCBI, USA). Subsequently, quality control and filtering of the raw reads were performed using Trimmomatic-0.39 [80]. The cleaned reads were then aligned to the sainfoin reference genome, and gene expression levels were quantified using StringTie v2.1.3 (GitHub, San Francisco, CA, USA) [81]. Gene expression levels are expressed as TPM (Transcripts Per Million) values [82]. Heat maps of gene expression were generated using TBtools software v2.326.

### 4.8. Plant Materials and Treatments

The common sainfoin cultivar used in this study was provided by the Key Laboratory of Xinjiang Phytomedicine Resources and Utilization, Ministry of Education, at Shihezi University (Shihezi, China). Seedlings were grown in a mixed substrate (nutrient soil: perlite: vermiculite = 3:1:1) in a plant growth chamber under controlled conditions: 25 °C, 16/8 h light/dark cycle, for 40 days. Subsequently, uniformly grown seedlings of consistent size were selected as experimental materials. These seedlings were first pre-cultured in Hoagland’s nutrient solution for 7 days for acclimation. Then, two treatments were applied: 50 μM MeJA and 100 μM ABA. Leaf, stem, and root samples were collected at 0 h (control), 3 h, 6 h, 12 h, and 24 h after treatment initiation. Each treatment included three biological replicates. The collected root, stem, and leaf samples were immediately frozen in liquid nitrogen and subsequently stored at −80 °C for subsequent experiments.

### 4.9. RT-qPCR Analysis

Total RNA was extracted from approximately 100 mg of the aforementioned frozen-ground plant tissue using the HiPure HP Plant RNA Mini Kit (Magen, Guangzhou, China). Subsequently, cDNA was synthesized from the RNA using a reverse transcription kit (Vazyme, Nanjing, China). Based on the coding sequence (CDS) of the target genes, specific primers were designed using Primer Premier software v5.0 (https://premierbiosoft.com/, accessed on 14 November 2024) (Supplementary Table S5). All primers were synthesized by Sangon Biotech Co., Ltd. (Shanghai, China). RT-qPCR reactions were performed on a LightCycler 480 II instrument (Roche, Shanghai, China). Each reaction was conducted with three biological replicates. The 20 µL reaction mixture consisted of 10 µL SYBR Green Master Mix (Vazyme, Nanjing, China), 0.6 µL of forward and reverse primers, 1.3 µL of diluted cDNA template, and 7.5 µL ddH_2_O. The thermal cycling protocol was as follows: 95 °C for 15 s, followed by 40 cycles of 95 °C for 10 s, 54–60 °C for 20 s, and 72 °C for 20 s; then 95 °C for 5 s, 65 °C for 1 min, and 40 °C for 30 s. The *OvActin* gene was used as the internal reference [83]. The relative expression levels of the target genes in different sainfoin tissues (roots, stems, leaves) were calculated based on the obtained Ct values using the 2^−∆∆Ct^ method [84]. All RT-qPCR experiments included three independent biological replicates. The experimental data are presented as the mean ± standard error of the mean (SEM). Data were statistically analyzed and visualized using GraphPad Prism software v9.5.1. Duncan’s multiple range test was employed for multiple comparisons, and differences were considered statistically significant at a probability level of *p* < 0.05.

### 4.10. Subcellular Localization

To determine the subcellular localization of OvANR proteins, an *Agrobacterium*-mediated transient expression assay in *Nicotiana benthamiana* leaves was employed. Seeds of *N. benthamiana*, provided by the Key Laboratory of Xinjiang Phytomedicine Resources and Utilization, Ministry of Education at Shihezi University (Shihezi, China), were used. The full-length coding sequences (CDS) of the *OvANR* genes were cloned into the plant expression vector pCAMBIA1300 via the BamHI and SalI restriction enzyme sites, generating the recombinant expression vectors pCAMBIA1300-35S-*OvANR*-eGFP. The empty vector pCAMBIA1300-35S-eGFP was constructed as a control. The cytoplasmic marker used was pCAMBIA1300-35S-mCherry-NOS (Puint, Xi’an, China). The constructed recombinant plasmids and the empty control vector were individually transformed into *Agrobacterium tumefaciens* strain GV3101. Single colonies were selected and cultured in LB liquid medium containing appropriate antibiotics at 28 °C with shaking at 200 rpm until the mid-logarithmic growth phase (OD_600_ ≈ 0.8–1.0). Bacterial cells were collected by centrifugation and resuspended in infiltration buffer to a final OD_600_ of 0.4–0.6. The suspensions were incubated at room temperature for 2–3 h before infiltration. Healthy, approximately 4-week-old *N. benthamiana* plants were selected, and fully expanded young leaves were used for infiltration. The prepared Agrobacterium suspensions were injected into the abaxial air spaces of the leaves using a needleless syringe. Following injection, the tobacco plants were kept in the dark at 25 °C for 24 h and then transferred to a light cycle (typically 16 h light/8 h dark) for an additional 48 h. Fluorescence signals were observed using a confocal laser scanning microscope (Nikon, Tokyo, Japan). GFP was excited at 488 nm, and mCherry was excited at 561 nm.

## 5. Conclusions

The *ANR* gene family is widely present in various plant species. In this study, we conducted the first genome-wide analysis of the *ANR* gene family in sainfoin, identifying a total of 67 *OvANR* genes. Subsequently, employing a range of bioinformatics approaches, we systematically analyzed the gene structures, conserved domains, conserved motifs, phylogenetic relationships, chromosomal locations, synteny, and cis-acting elements of this family, revealing its evolutionary characteristics and regulatory potential. Combined with tissue-specific expression patterns, *OvANR19* was found to be significantly upregulated in roots and leaves following ABA and MeJA treatments. Furthermore, its promoter is enriched with stress-responsive elements (e.g., ABRE, G-box), suggesting a potential core role for this gene in stress resistance. Additionally, subcellular localization results indicated that ANR proteins likely perform their biological functions in the cytoplasm. This study not only provides the first systematic characterization of the *ANR* gene family in sainfoin but also establishes a crucial foundation for future functional validation of these genes and for elucidating the biosynthesis of proanthocyanidins and their underlying mechanisms of action.

## Data Availability

The original contributions presented in this study are included in the article/Supplementary Material. Further inquiries can be directed to the corresponding authors.

## Supplementary Materials

The following supporting information can be downloaded at: https://www.mdpi.com/article/10.3390/ijms262311256/s1.
