# Supplementary material for: Genome-Wide Identification and Hormone-Induced Expression Analysis of the Anthocyanidin Reductase Gene Family in Sainfoin (Onobrychis viciifolia Scop.)
Source: Int J Mol Sci. 2025 Nov 21;26(23):11256. doi: 10.3390/ijms262311256 (PMC12691743; doi:10.3390/ijms262311256)
Supplement: Supplementary file 1 [file ijms-26-11256-s001.zip › Figure S1.pdf]

Figure S1. OvANR gene family protein tertiary structure.

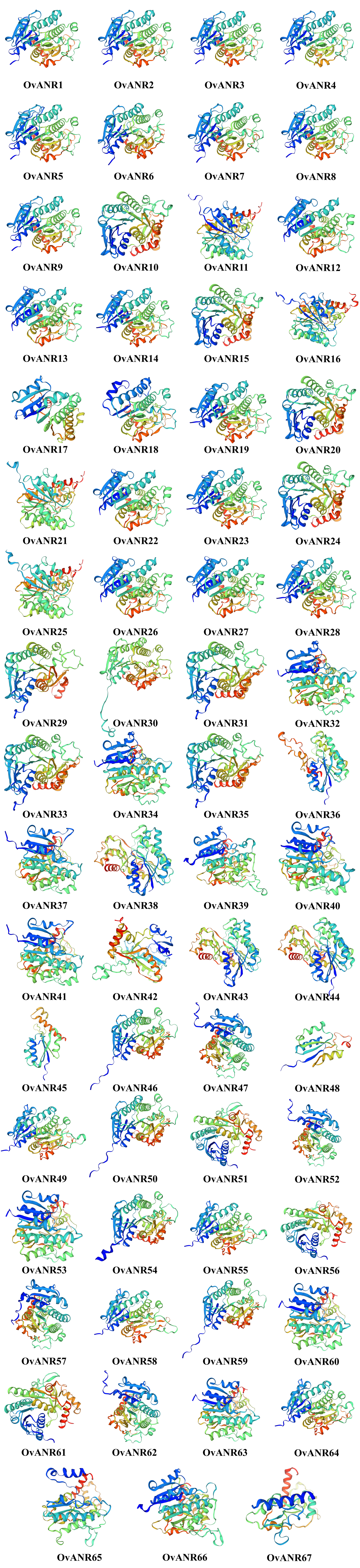

Figure S1. OvANR gene family protein tertiary structure. Structures were modeled using Swiss-Model and are displayed as rainbow-colored ribbon diagrams (N-terminus = red, C-terminus = blue).
