# Supplementary material for: Genome-Wide Identification and Hormone-Induced Expression Analysis of the Anthocyanidin Reductase Gene Family in Sainfoin (Onobrychis viciifolia Scop.)
Source: Int J Mol Sci. 2025 Nov 21;26(23):11256. doi: 10.3390/ijms262311256 (PMC12691743; doi:10.3390/ijms262311256)
Supplement: Supplementary file 1 [file ijms-26-11256-s001.zip › Figure S3.pdf]

Figure S3. Expression profiles of the *ANR* gene in *Onobrychis viciifolia* under cold and drought stress.

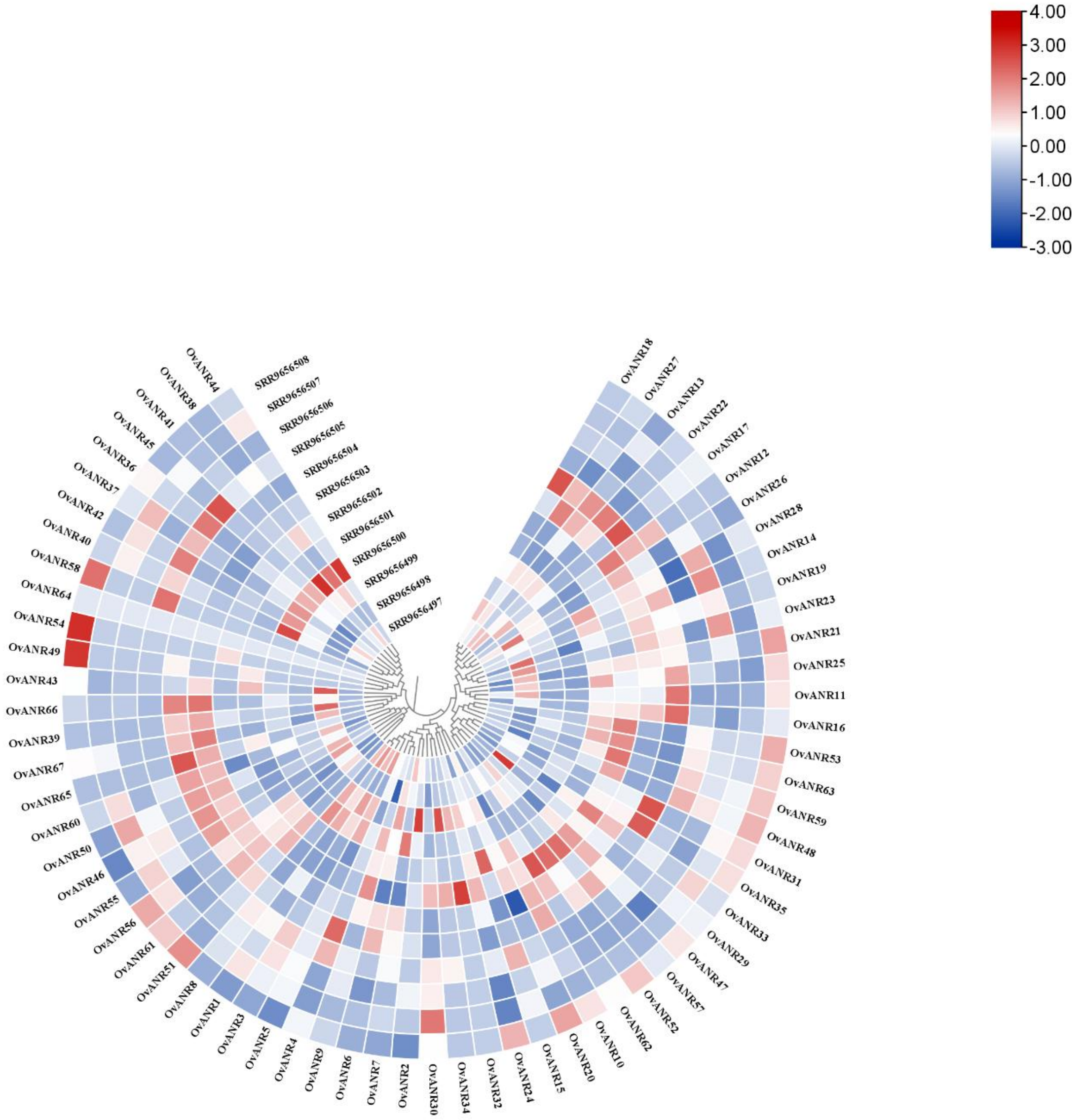

Figure S3. Expression profiles of the *ANR* gene in *Onobrychis viciifolia* under cold and drought stress. The color bar from red to blue indicates relative expression levels from higher to lower, respectively.
