# Supplementary material for: Genome-Wide Identification and Hormone-Induced Expression Analysis of the Anthocyanidin Reductase Gene Family in Sainfoin (Onobrychis viciifolia Scop.)
Source: Int J Mol Sci. 2025 Nov 21;26(23):11256. doi: 10.3390/ijms262311256 (PMC12691743; doi:10.3390/ijms262311256)
Supplement: Supplementary file 1 [file ijms-26-11256-s001.zip › Figure S4.pdf]

Figure S4. Expression profiles of eight *OvANR* genes in *Onobrychis viciifolia* stems under ABA and MeJA treatments.

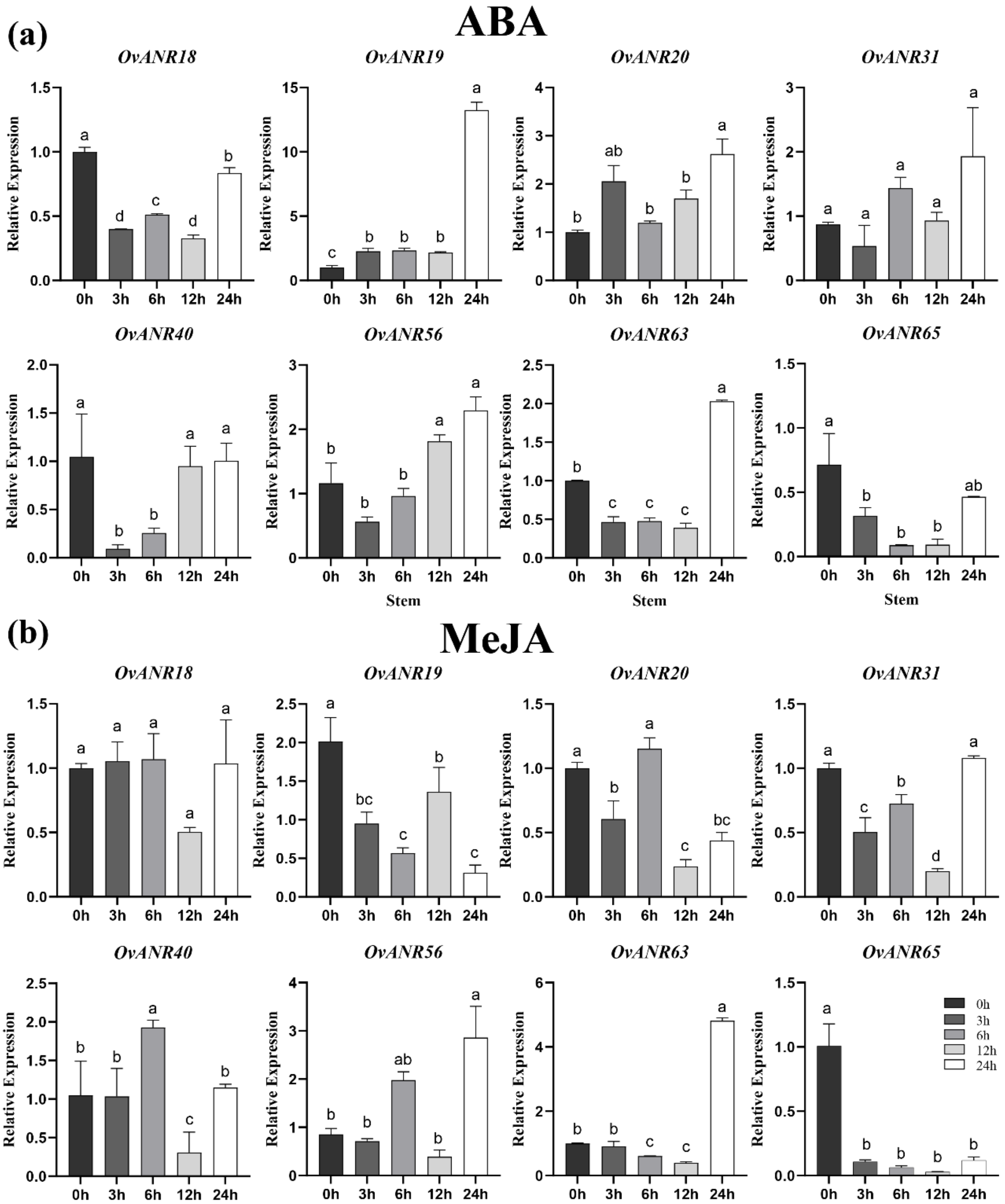

Figure S4. Expression profiles of eight *OvANR* genes in *Onobrychis viciifolia* stems under ABA and MeJA treatments. (a) Expression after ABA treatment at 0 h (control), 3 h, 6 h, 12 h, and 24 h. (b) Expression after MeJA treatment at 0 h (control), 3 h, 6 h, 12 h, and 24 h. Data were normalized using the *Actin* gene as an internal reference. Error bars represent standard deviations. Different letters above bars indicate statistically significant differences ($p<0.05$) as determined by Duncan's multiple range test.
