# Supplementary material for: Genome-Wide Identification and Hormone-Induced Expression Analysis of the Anthocyanidin Reductase Gene Family in Sainfoin (Onobrychis viciifolia Scop.)
Source: Int J Mol Sci. 2025 Nov 21;26(23):11256. doi: 10.3390/ijms262311256 (PMC12691743; doi:10.3390/ijms262311256)
Supplement: Supplementary file 1 [file ijms-26-11256-s001.zip › Table S2.pdf]

Table S2. Secondary structure predictions of OvANR proteins.

Gene Name	α-Helix (%)	β-Turn (%)	Extended Strand(%)	Random Coil(%)	Structures
OvANR1	29.20	12.35	22.84	25.62	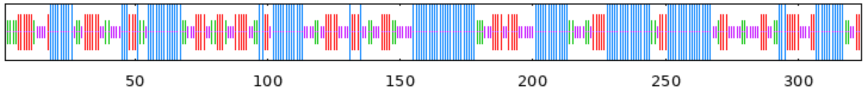
OvANR2	44.48	10.43	23.62	21.47	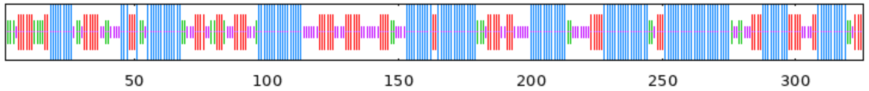
OvANR3	38.27	12.04	22.84	26.85	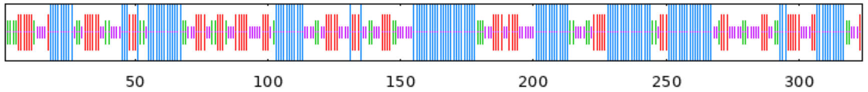
OvANR4	37.67	9.21	23.31	29.81	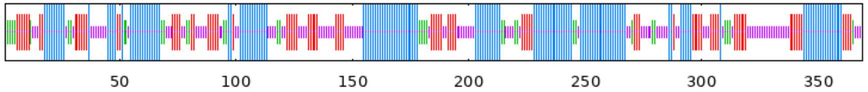
OvANR5	41.79	9.25	22.39	26.57	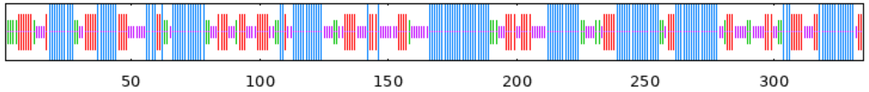
OvANR6	45.15	8.18	20.61	16.06	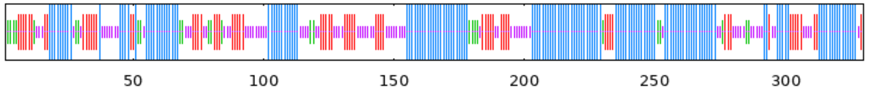
OvANR7	40.49	10.74	23.01	25.77	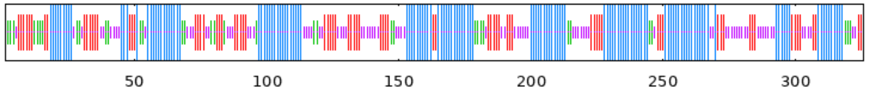
OvANR8	38.27	12.04	23.15	26.54	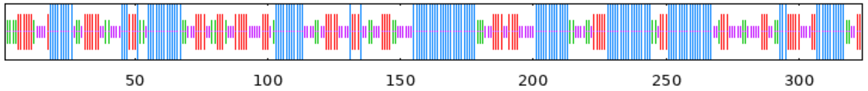
OvANR9	41.98	8.95	21.30	27.78	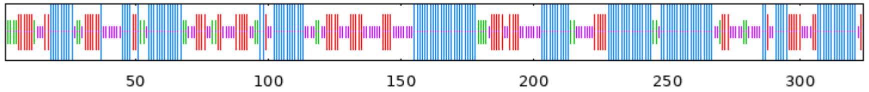
OvANR10	42.32	9.09	17.24	31.35	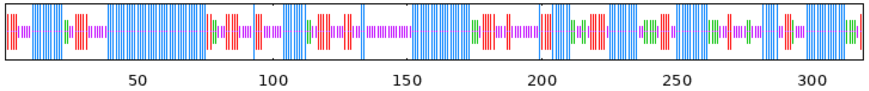

OvANR11	33.03	11.11	19.52	36.34	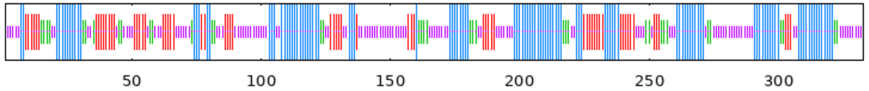
OvANR12	50.94	9.06	13.75	26.25	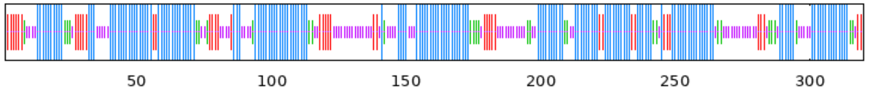
OvANR13	47.19	10.31	15.00	27.50	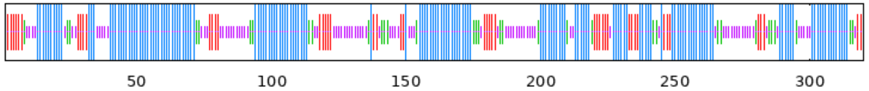
OvANR14	52.35	9.40	15.05	23.20	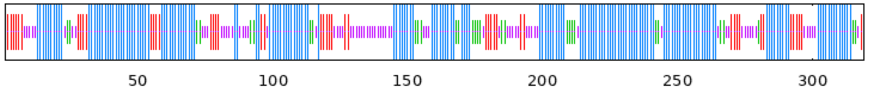
OvANR15	42.32	8.15	16.61	32.92	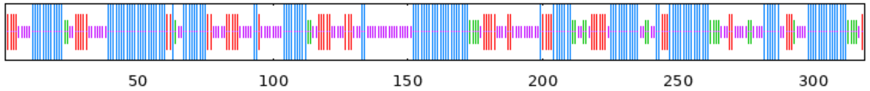
OvANR16	32.39	9.94	20.17	37.50	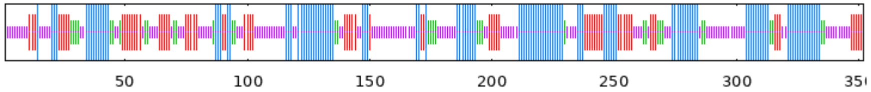
OvANR17	47.91	6.98	14.88	30.23	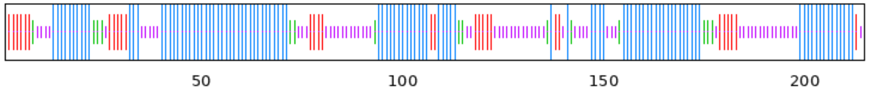
OvANR18	46.36	10.73	13.03	29.89	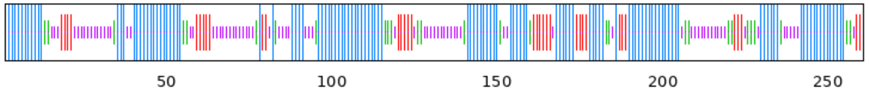
OvANR19	52.35	9.09	15.36	23.20	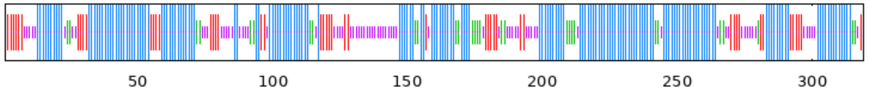
OvANR20	40.75	9.40	17.55	32.29	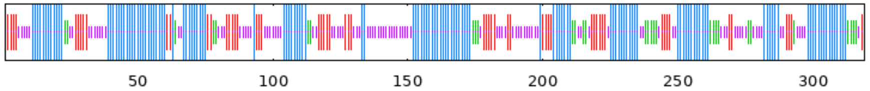
OvANR21	33.49	10.85	19.17	36.49	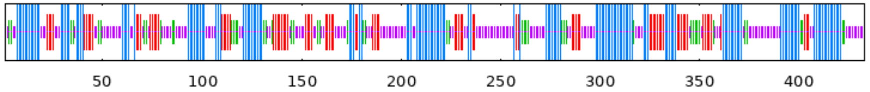

OvANR22	47.50	10.00	15.31	27.19	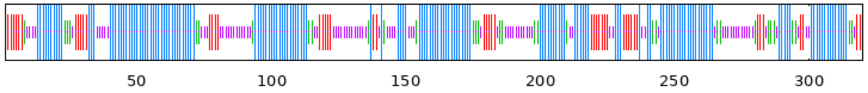
OvANR23	52.35	9.09	15.36	23.20	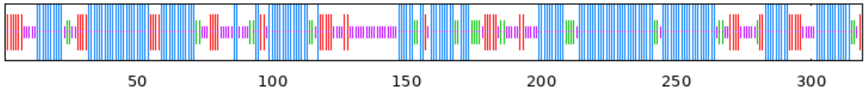
OvANR24	40.44	8.46	17.55	33.54	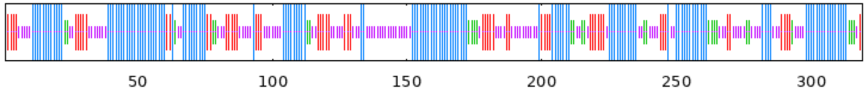
OvANR25	33.72	10.62	18.71	36.95	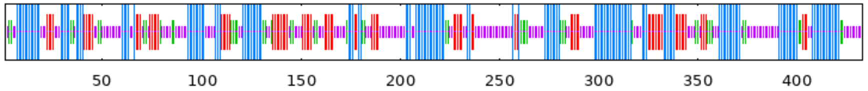
OvANR26	50.94	9.06	12.81	27.19	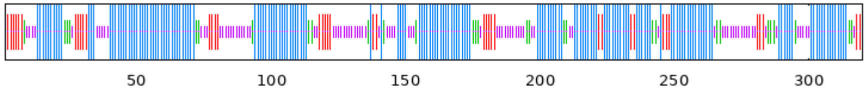
OvANR27	47.50	10.31	13.75	28.44	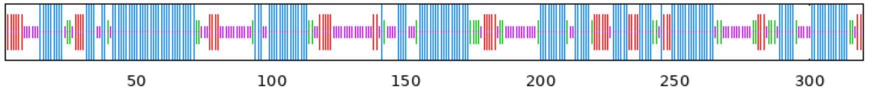
OvANR28	52.98	9.72	14.42	22.88	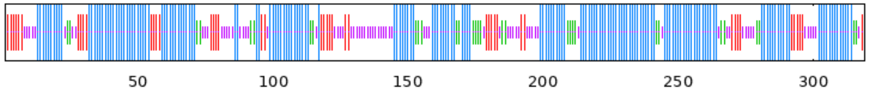
OvANR29	42.61	7.90	20.62	28.87	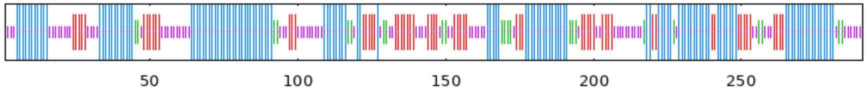
OvANR30	37.10	7.54	17.86	37.50	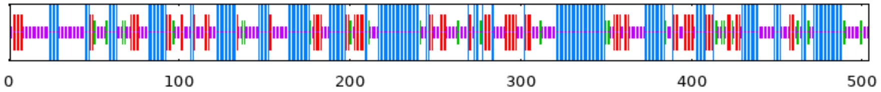
OvANR31	43.06	8.38	18.21	30.35	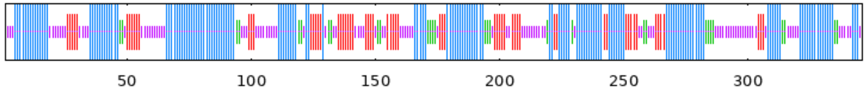
OvANR32	39.33	8.54	20.12	32.01	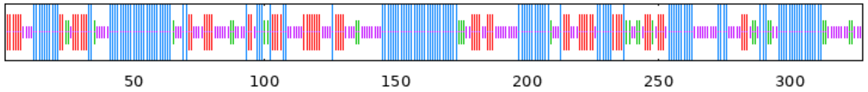

OvANR33	42.69	7.89	18.13	31.29	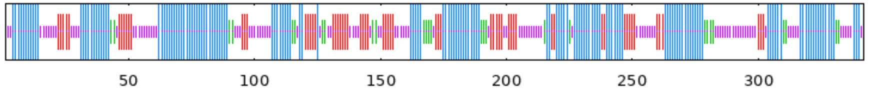
OvANR34	39.33	8.54	20.12	32.01	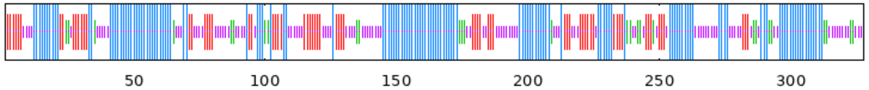
OvANR35	41.64	8.50	18.48	31.38	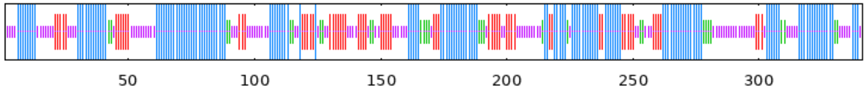
OvANR36	34.27	27.02	11.29	27.42	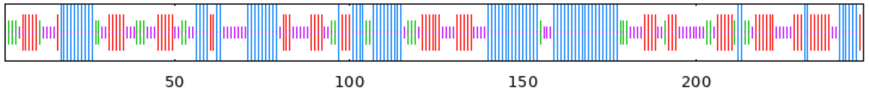
OvANR37	39.26	10.12	21.78	28.83	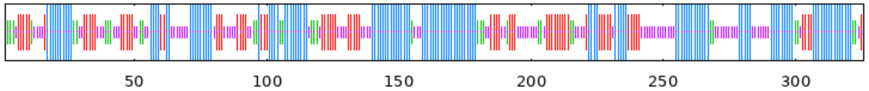
OvANR38	30.06	11.96	26.07	31.90	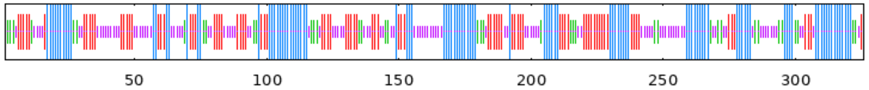
OvANR39	38,41	11.59	21.34	28.66	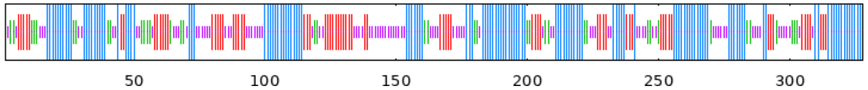
OvANR40	40.80	9.20	20.55	29.45	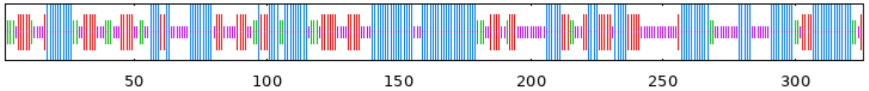
OvANR41	33.62	9.60	24.58	32.20	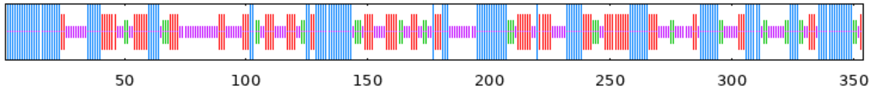
OvANR42	37,12	9.51	23.01	30.37	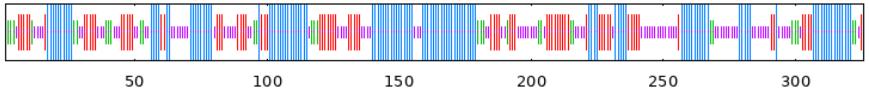
OvANR43	27.57	9.97	26.25	36.21	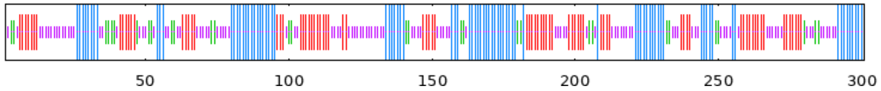

OvANR44	30.06	11.04	26.07	32.82	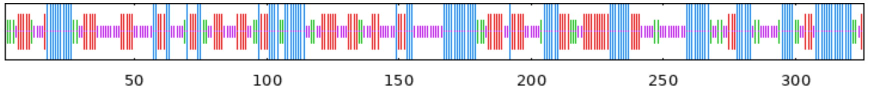
OvANR45	29.08	11.35	24.82	34.75	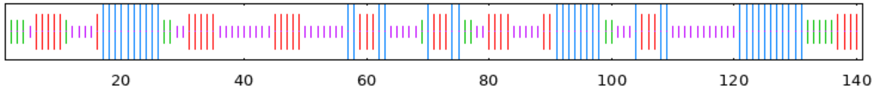
OvANR46	41.89	8.55	15.34	34.22	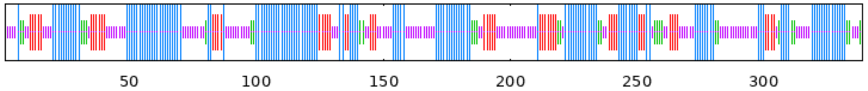
OvANR47	35.05	8.16	19.34	37.46	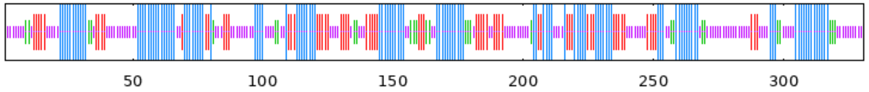
OvANR48	43.43	8.08	23.23	25.25	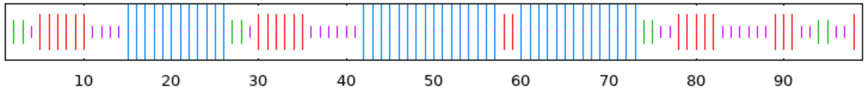
OvANR49	43.88	11.34	16.72	28.06	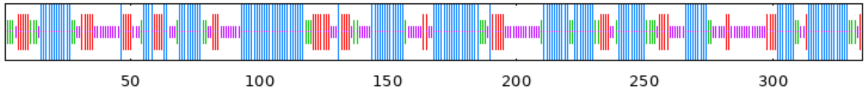
OvANR50	41.89	8.55	15.34	34.22	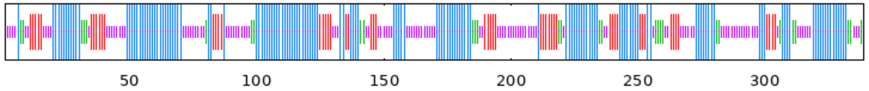
OvANR51	40.65	6.53	16.32	36.50	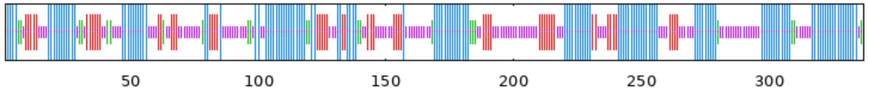
OvANR52	35.35	7.55	19.34	37.76	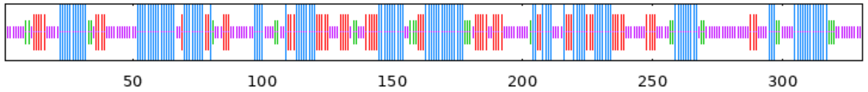
OvANR53	48.75	8.12	20.00	23.12	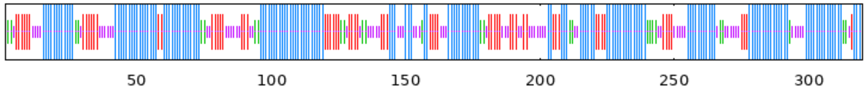
OvANR54	43.88	11.34	16.72	28.06	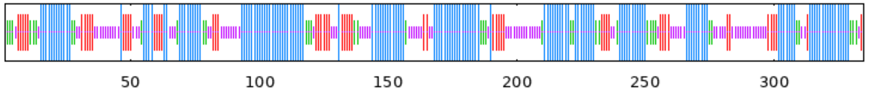

OvANR55	40.71	8.85	15.63	34.81	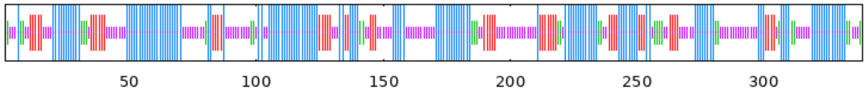
OvANR56	42.72	8.23	16.14	32.91	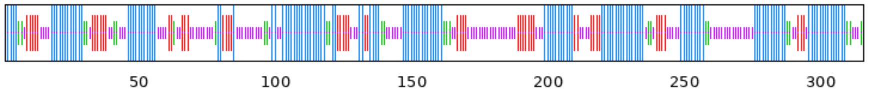
OvANR57	35.05	8.16	19.34	37.46	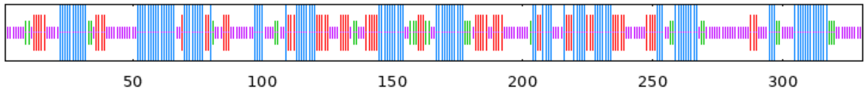
OvANR58	40.33	10.16	19.02	30.49	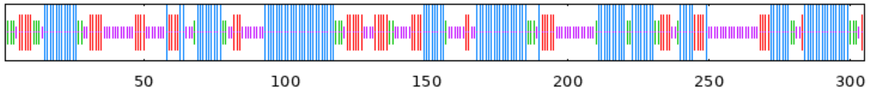
OvANR59	47.81	7.81	20.00	24.38	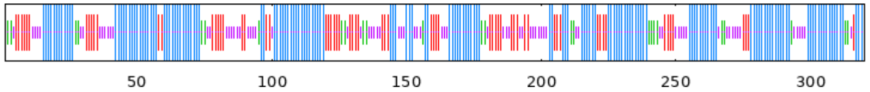
OvANR60	41.89	8.55	15.34	34.22	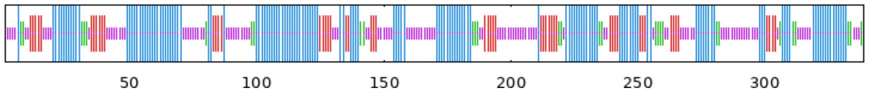
OvANR61	40.65	7.72	15.73	35.91	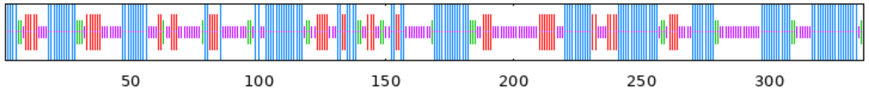
OvANR62	32.75	8.70	20.87	37.68	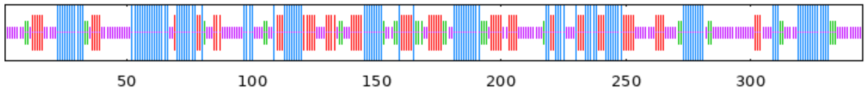
OvANR63	48.75	8.12	19.69	23.44	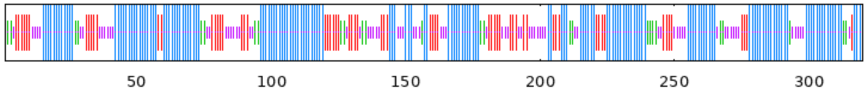
OvANR64	43.28	11.64	17.01	28.06	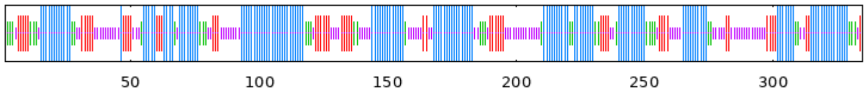
OvANR65	39.45	10.73	20.07	29.76	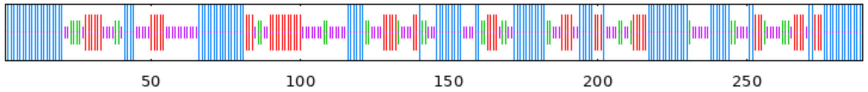

OvANR66	36.75	11.40	21.65	30.20	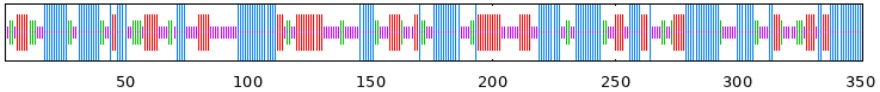
OvANR67	46.24	11.56	18.50	23.70	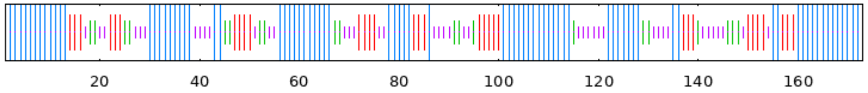

Table S2. Secondary structure predictions of OvANR proteins. The secondary structure prediction of OvANR proteins indicated that they are primarily composed of α-helices, β-sheets, extended strands, and random coils.
