# Supplementary material for: Genome-Wide Identification and Hormone-Induced Expression Analysis of the Anthocyanidin Reductase Gene Family in Sainfoin (Onobrychis viciifolia Scop.)
Source: Int J Mol Sci. 2025 Nov 21;26(23):11256. doi: 10.3390/ijms262311256 (PMC12691743; doi:10.3390/ijms262311256)
Supplement: Supplementary file 1 [file Figure S5.pdf › Figure S5.pdf]

Figure S5. Multiple sequence alignment of OvANR proteins.

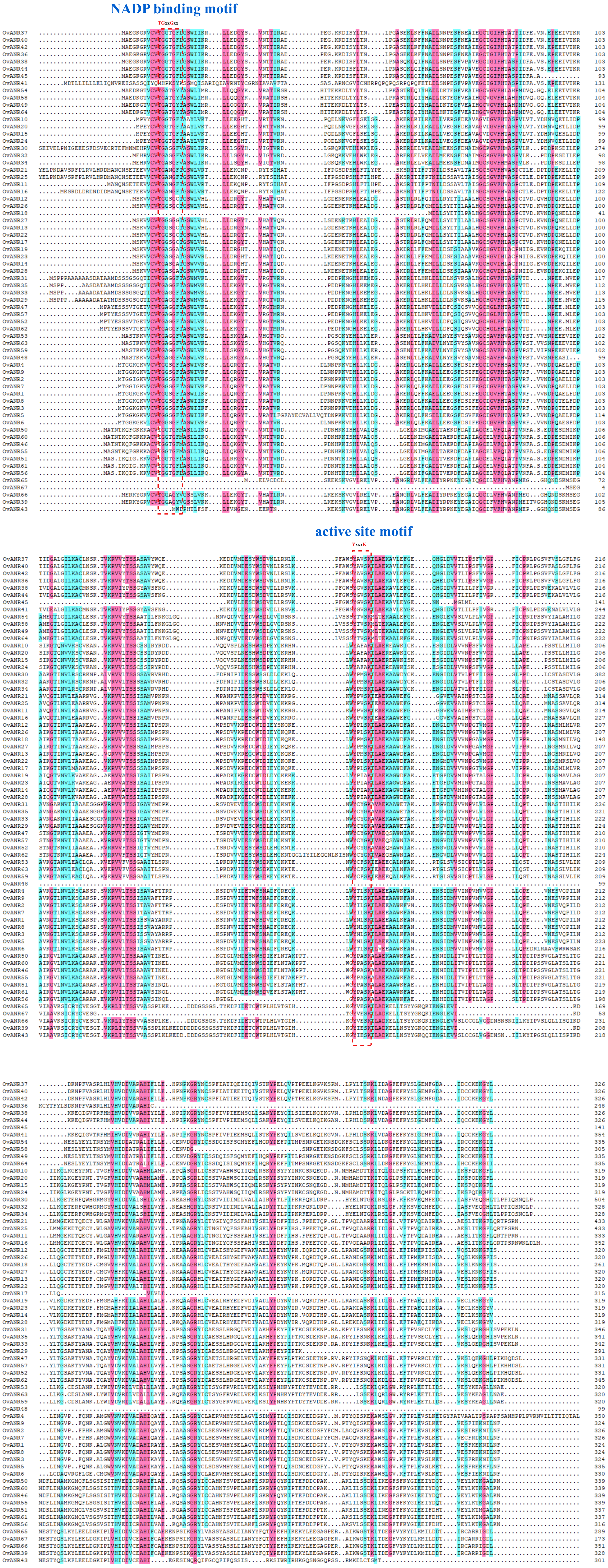

Figure S5. Multiple sequence alignment of OvANR proteins. The conservation of residues is indicated by background colors: pink for highly conserved (identity > 70%) and blue for moderately conserved (identity > 50%). Functional motifs are highlighted by boxes: red for the NADP-binding site and green for the active site. Residues are colored according to their amino acid properties.
